# Field validation of clinical and laboratory diagnosis of wildebeest associated malignant catarrhal fever in cattle

**DOI:** 10.1186/s12917-019-1818-8

**Published:** 2019-02-28

**Authors:** Sheillah Ayiela Orono, George Chege Gitao, Jean Pierre Mpatswenumugabo, Maurine Chepkwony, Christine Mutisya, Edward Okoth, Barend Mark de Clare Bronsvoort, George Cameron Russell, Vishvanath Nene, Elizabeth Anne Jessie Cook

**Affiliations:** 1 Department of Veterinary Pathology, Microbiology and Parasitology, University of Nairobi, P. O. Box, 29053, Kangemi, Kenya; 2 International Livestock Research Institute, Old Naivasha Road, P. O. Box 30709, Nairobi, Kenya; 3 Department of Veterinary Medicine, University of Rwanda, P.O. Box: 210, Musanze, Rwanda; 4 The Roslin Institute, Royal (Dick) School of Veterinary Studies, University of Edinburgh, Roslin, Midlothian, EH25 9RG UK; 5 Moredun Research Institute, Penicuik, Midlothian, EH26 0PZ UK

**Keywords:** Herpesvirus, Kenya, Wildebeest associated malignant catarrhal fever, No-gold standard

## Abstract

**Background:**

Wildebeest associated malignant catarrhal fever (WA-MCF) is a fatal disease of cattle. Outbreaks are seasonal and associated with close interaction between cattle and calving wildebeest. In Kenya, WA-MCF has a dramatic effect on cattle-keepers who lose up to 10% of their cattle herds per year. The objective of this study was to report the impact of WA-MCF on a commercial ranch and assess the performance of clinical diagnosis compared to laboratory diagnosis as a disease management tool.

A retrospective study of WA-MCF in cattle was conducted from 2014 to 2016 at Kapiti Plains Ranch Ltd., Kenya. During this period, 325 animals showed clinical signs of WA-MCF and of these, 123 were opportunistically sampled. In addition, 51 clinically healthy animals were sampled. Nested polymerase chain reaction (PCR) and indirect enzyme linked immunosorbent assay (ELISA) were used to confirm clinically diagnosed cases of WA-MCF. A latent class model (LCM) was used to evaluate the diagnostic parameters of clinical diagnosis and the tests in the absence of a gold standard.

**Results:**

By PCR, 94% (95% C.I. 89–97%) of clinically affected animals were positive to WA-MCF while 63% (95% C.I. 54–71%) were positive by indirect ELISA. The LCM demonstrated the indirect ELISA had poor sensitivity 63.3% (95% PCI 54.4–71.7%) and specificity 62.6% (95% PCI 39.2–84.9%) while the nested PCR performed better with sensitivity 96.1% (95% PCI 90.7–99.7%) and specificity 92.9% (95% PCI 76.1–99.8%). The sensitivity and specificity of clinical diagnosis were 99.1% (95% PCI 96.8–100.0%) and 71.5% (95% PCI 48.0–97.2%) respectively.

**Conclusions:**

Clinical diagnosis was demonstrated to be an effective method to identify affected animals although animals may be incorrectly classified resulting in financial loss. The study revealed indirect ELISA as a poor test and nested PCR to be a more appropriate confirmatory test for diagnosing acute WA-MCF. However, the logistics of PCR make it unsuitable for field diagnosis of WA-MCF. The future of WA-MCF diagnosis should be aimed at development of penside techniques, which will allow for fast detection in the field.

**Electronic supplementary material:**

The online version of this article (10.1186/s12917-019-1818-8) contains supplementary material, which is available to authorized users.

## Background

Malignant catarrhal fever (MCF) is a fatal viral illness of cattle [1]. The causative viruses of MCF belong to subfamily *Gammaherpesvirinae*, [2] genus *Macavirus* [3]. Worldwide there are two main viruses responsible for MCF in cattle; these are alcelaphine herpesvirus 1 (AlHV-1), resulting in wildebeest associated malignant catarrhal fever (WA-MCF), and ovine herpesvirus 2 (OvHV-2), resulting in sheep (*Ovies aries*) associated malignant catarrhal fever (SA-MCF). WA-MCF primarily occurs in sub-Saharan Africa and is confined to geographical regions where cattle graze with wildebeest [4]. Both blue and black wildebeest (*Connochaetes testaurinus* and *C. gnou* respectively) exist as natural hosts for AlHV-1 [4].

There are yearly epidemics of WA-MCF in Kenya that normally coincide with the wildebeest calving season [4] with peak incidence occurring between March and June [5]. The south western region of Kenya forms the location of the three major wildebeest areas [4]. These are the Maasai Mara ecosystem, including the Maasai Mara National Reserve, extending into the Serengeti in Tanzania; the Athi-Kaputiei environment including the Nairobi National Park, and the Athi-Kaputiei plains; and the Amboseli-Kilimanjaro ecosystem including the Amboseli National Park and extending into Mt. Kilimanjaro in Tanzania [6–8]. In the field, diagnosis is by clinical signs, which include oculonasal discharge, sudden fever, corneal opacity, swollen lymph nodes, conjunctivitis and erosive mucosal lesions in the upper respiratory tract [9]. Differential diagnoses include bovine viral diarrhea (BVD)/mucosal disease, rinderpest, foot and mouth disease (FMD), bluetongue and vesicular stomatitis. Laboratory diagnosis is confirmed by positive serology or PCR [10]. The definitive diagnoses are confirmed through post mortem histopathological analysis of samples from dead cattle.

Several diagnostic tests for the detection of antibodies to MCF viruses have been described [11]. These assays use AlHV-1 as the antigen since this virus can be propagated in vitro [11]**.** Serological tests used to identify AlHV-1 infection include indirect ELISA [12, 13], competitive inhibition (CI)- ELISA [14–16], virus neutralization test (VNT) [12, 17] and indirect fluorescent antibody test [18]. The indirect ELISA depends on a polyclonal response, which may be more robust in detecting sick animals with partial or low antibody titres compared with the CI-ELISA [13]. In MCF-susceptible hosts like cattle, no virus-neutralizing antibody response is induced, hence antibodies are detected using ELISA, indirect immunofluorescence or Western blot [10].

An important tool for the molecular diagnosis of MCF is PCR [11]. The first PCR assay for detecting AlHV-1 deoxyribonucleic acid (DNA) was described in 1990 [19] and since then several DNA based assays have been developed [11]. These can differentiate AlHV-1 from other macaviruses and have higher diagnostic sensitivity than ELISA assays for diagnosis of MCF [4]. DNA based assays previously used for the detection of AlHV-1 include nested PCR [20, 21], consensus PCR [11] and real time PCR [22, 23] .

The World Organization for Animal Health (OIE) no longer classifies WA-MCF as a trans boundary or notifiable disease, however it is of importance in Kenya in regions where cattle co-graze with wildebeest. WA-MCF is viewed as a significant cattle illness in sub Saharan Africa with the highest impact on the livelihoods of cattle-keeping communities [24]. The disease has direct impacts on both commercial and smallholder farmers whose economic livelihoods are affected by the death of their cattle and additionally the loss of income from milk and beef sales [24, 25].

There is currently no vaccine or treatment available for WA-MCF [4]. The current control method for WA-MCF in affected regions is preventing interaction between cattle and wildebeest carriers [4, 5]. Cattle owners attempt to reduce losses through disposal of sick cattle or through disease avoidance strategies [4]. WA-MCF affected cattle will be sold at less than 50% of the market value of healthy cattle [5, 24]. Avoidance strategies mean that cattle-keepers graze their animals on poorer pastures away from wildebeests [4]. The energy requirements during cattle movement over hundreds of kilometers and poor quality of alternative pastures reduce milk production and affect body condition reducing market value of cattle [25]. There are also labour and travel related costs of avoiding WA-MCF with family members spending up to three months away from home [25].

The annual incidence of WA-MCF in Kenya is estimated to be 3 to 12% [24]. The absence of a simple and cost effective diagnostic test for AlHV-1 may contribute to underreporting and misdiagnosis of WA-MCF cases and may explain the high variability in the reported annual WA-MCF incidence rates in sub-Saharan Africa [4]. Currently cattle keepers clinically diagnose animals with WA-MCF without laboratory confirmation [5]. The objective of this study was to report the impact of WA-MCF on a commercial ranch and assess the performance of clinical diagnosis compared to laboratory diagnosis as a disease management tool.

## Results

### Descriptive results

#### 2014 WA-MCF outbreak

**Fig. 1:**
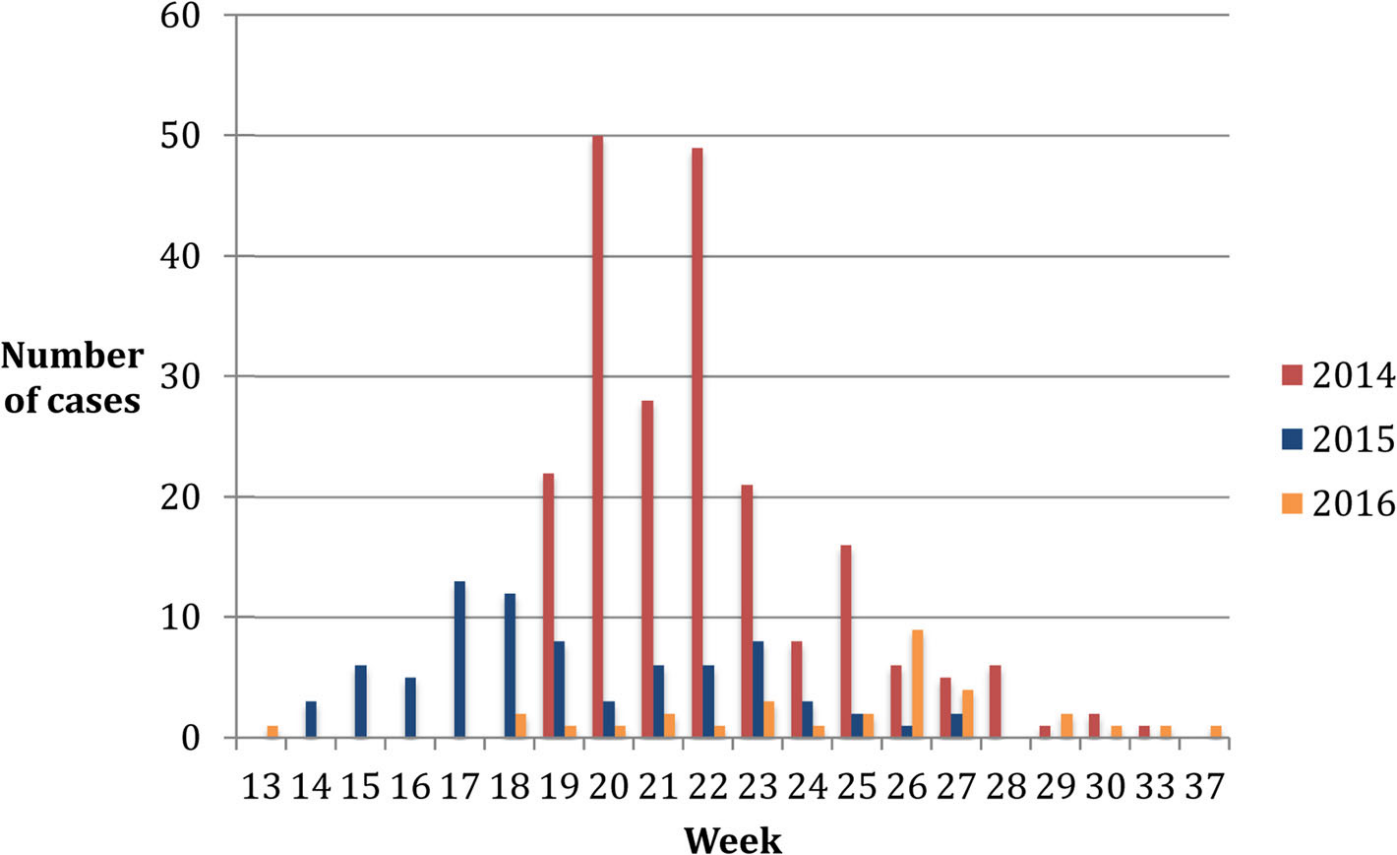
Epidemic curve of malignant catarrhal fever at Kapiti Plains Ranch, 2014–2016 demonstrating the number of cases per week in each year studied

The 2014 WA-MCF outbreak at Kapiti Plains Ranch started on May 7th (week 19 of 2014) and ended July 31st in (week 31) affecting 215 animals (Fig. 1). The herd size at the beginning of the outbreak was 2467 animals. The incidence of WA-MCF in 2014 was 8.7 (95% C.I. 7.6–9.9) cases per hundred animals per year (Additional file 1). The estimated direct losses to Kapiti Plains Ranch were estimated ~ US$64,500.

There was no significant difference in WA-MCF incidence between sexes, OR 1.00 (95% C.I. 0.71–1.37). However, there was a significant difference in WA-MCF incidence between age groups with steers, heifers and cows more likely to have WA-MCF compared to calves. The OR for steers was 4.53 (95% C.I. 2.29–10.03), heifers 3.85 (95% C.I. 2.00–8.37), and cows 2.79 (95% C.I. 1.44–6.07) respectively (Additional file 2).

#### 2015 WA-MCF outbreak

The 2015 outbreak started on April 2nd (week 14 of 2015) and ended June 30th (week 27) involving 78 animals (Fig. 1). The herd size at the beginning of the outbreak was 2106. The incidence in 2015 was 3.7 (95% C.I. 3.0–4.6) cases per hundred animals per year (Additional file 1). The estimated direct losses to Kapiti Plains Ranch were estimated ~US$23,400.

There was a significant difference in WA-MCF incidence between sexes, with males having an OR of 0.38 (95% C.I. 0.16–0.78) compared to females. There was no significant difference between the incidence of WA-MCF cases within the age groups although there was a positive relationship with heifers and cows more likely to have WA-MCF compared to calves. The OR for heifers was 1.72 (95% C.I. 0.86–3.64) and cows 1.49 (95% C.I. 0.76–3.11) respectively (Additional file 2).

#### 2016 WA-MCF outbreak

The outbreak in 2016 started on March 29th (week 13 of 2016) and ended September 19th (week 37) involving 32 animals (Fig. 1). The herd size at the beginning of the outbreak was 2069 animals. The incidence of WA-MCF in 2016 was 1.5 (95% C.I. 1.1–2.2) cases per hundred animals per year (Additional file 1). The estimated direct losses to Kapiti Plains Ranch were estimated ~US$9600.

There was no significant difference between sexes with respect to WA-MCF incidence, males having an OR 0.45 (95% C.I. 0.13–1.15). There was a positive but not a significant relationship between age group and WA-MCF incidence with cows and steers being more likely to have WA-MCF compared to calves, OR 2.98 (95% C.I. 0.88–18.60) and 2.29 (95% C.I. 3.7–17.57) respectively (Additional file 2).

### Laboratory results

A total of 123 samples were collected from 325 clinically affected animals between 2014 and 2016. Samples were tested using nested PCR and indirect ELISA. Of the 123 clinically diagnosed samples, 116 samples were positive by nested PCR, while 77 were positive by indirect ELISA (Table 1). Fifty-one samples were collected from clinically healthy animals in 2016. Of these, none were positive by nested PCR, while 3 were positive by indirect ELISA (Table 1). The individual results for each animal are available in Additional file 3.

**Table 1:** Overall results of PCR and ELISA for WA-MCF cases and clinically negative animals collected between 2014 and 2016

	Clinically positive		Clinically negative		All samples	
	PCR positive	PCR negative	PCR positive	PCR negative	PCR positive	PCR negative
ELISA positive	74	3	0	3	74	6
ELISA negative	42	4	0	48	42	52
Total	116	7	0	51	116	58

Using all samples, a comparison of indirect ELISA to nested PCR as the gold standard revealed its sensitivity and specificity to be 63.8% (95% C.I. 54.4–72.5%) and 89.7% (95% C.I. 78.8–86.1%) respectively (Table 2). A kappa value of 0.47 was obtained which revealed moderate agreement between these two tests.

**Table 2:** Diagnostic test parameters for indirect ELISA using nested PCR as a gold standard

Parameter	Result	95% C.I
Sensitivity	63.8	54.4–72.5
Specificity	89.7	78.8–96.1
Positive Likelihood Ratio (PLR)	6.2	2.9–13.3
Negative Likelihood Ratio (NLR)	0.4	0.3–0.5
Positive Predictive Value (PPV)	92.5%	85.1–96.4
Negative Predictive Value (NPV)	55.3%	48.9–61.6

### Bayesian Agreement Index (BAI)

The diagnostic parameters of both indirect ELISA and nested PCR for all samples were evaluated in the absence of a gold standard using the BAI. This revealed agreement for these two assays to be better in the positive than in the negative direction with a BAI of 75.5% (95% BCI 69.0–82.0%) in the positive agreement and 68.4% (95% BCI 59.0–76.0%) in the negative direction.

### Latent class model

**Fig. 2:**
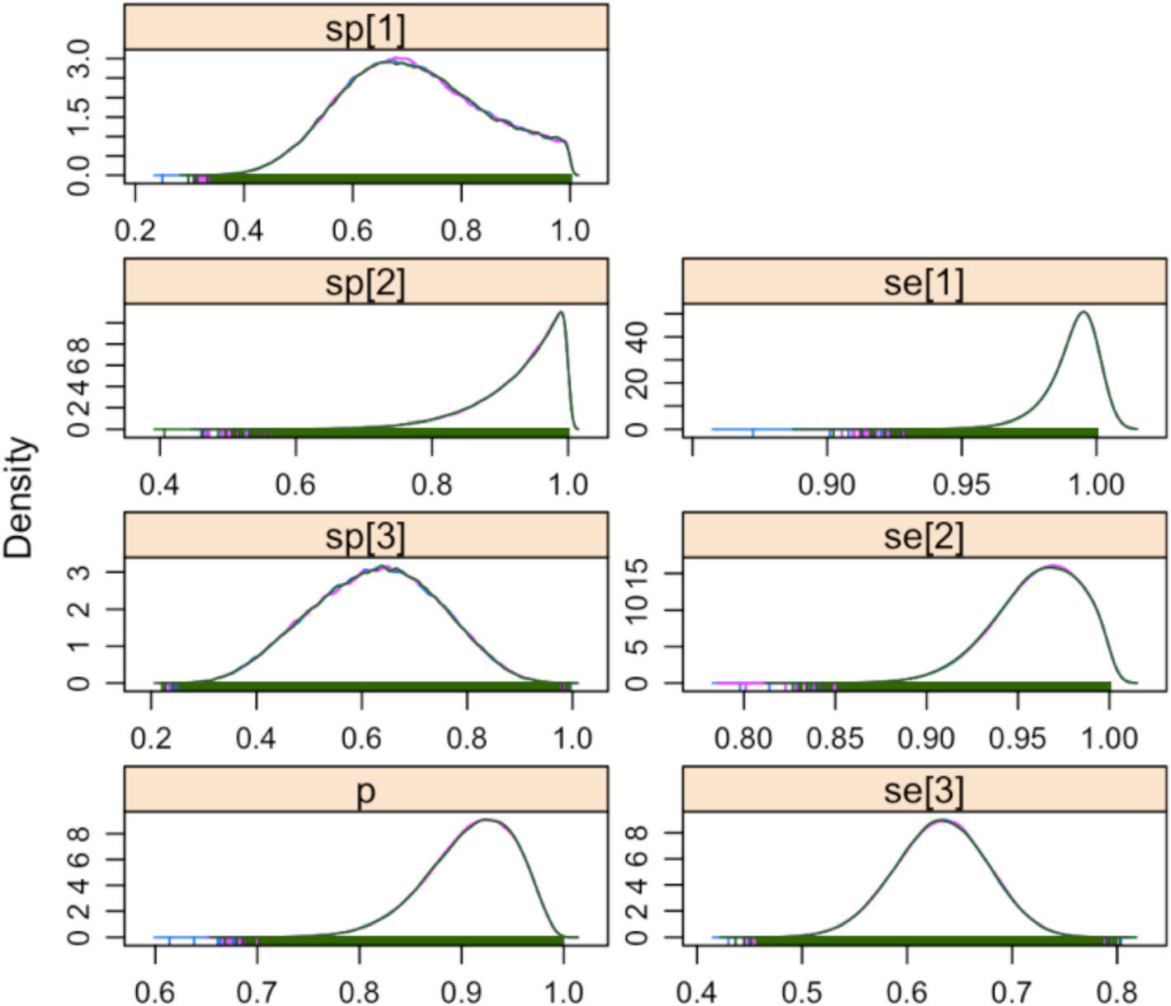
Posterior Probability Density Plots for Estimated Parameters, Se/Sp [1] = Clinical diagnosis, Se/Sp [2] = PCR, Se/Sp [3] = ELISA, p = sample prevalence

The posterior estimates of sensitivity and specificity for each diagnostic are given in Table 3 and the distributions are plotted in Fig. 2. The results suggest that the indirect ELISA is less appropriate as a diagnostic test in clinically ill animals, presumably because they may have not mounted an antibody response, with a sensitivity of 63.3% (95% PCI 54.4–71.1%) and specificity of 62.6% (95% PCI 39.2–84.9%) while the nested PCR is more appropriate with a sensitivity of 96.1% (95% PCI 90.7–99.7%) and specificity of 92.9% (95% PCI 76.1–99.8%). The sensitivity and specificity of clinical diagnosis was 99.1% (95% PCI 96.8–100.0%) and 71.5% (95% PCI 48.0–97.2%), respectively.

**Table 3:** Posterior Estimations of Diagnostic Test Parameters with 95% Bayesian Credibility Intervals

Parameter	Posterior estimate % (95% PCI)
Sensitivity ELISA	63.3 (54.4–71.7)
Sensitivity PCR	96.1 (90.7–99.7)
Sensitivity Clinical diagnosis	99.1 (96.8–100.0)
Specificity ELISA	62.6 (39.3–84.9)
Specificity PCR	92.9 (76.1–99.8)
Specificity Clinical Diagnosis	71.5 (48.0–97.2)

## Discussion

The outbreaks of WA-MCF in 2014 and 2015 occurred between the months of April and July. This coincides with the period of the highest transmission of WA-MCF in Kenya reported between March and June [4]. The outbreak in 2016 was the longest and occurred between the months of March to September. Changes in rainfall and pasture availability are likely to affect wildebeest calving seasons and this may explain the variation in the timing of outbreaks. Increased stress on wildebeest through pregnancy or nutritional deficits can also result in longer periods of transmission [4]. In addition, outbreaks that occur from September to November in Kenya may be due to cattle undergoing stress because of cold weather in the “winter” period (June–August) and increased susceptibility to infection [4].

The incidence of WA-MCF in 2014 was 8.7% and in 2015 was 3.7%. This agrees with previous estimates of the incidence of WA-MCF in Kenya to be 3–12% [24]. The reduction in incidence between 2014 and 2015 was likely due to the implementation of wildebeest avoidance measures to reduce contact between cattle and wildebeest on the ranch, including fencing and actively grazing cattle away from wildebeest herds. The incidence in 2016 (1.5%) was less than previous years and this reflects the lower numbers of wildebeest observed on the ranch in 2016. The reduced numbers of wildebeest on the ranch in 2016 may have been the result of drought [26]. Previous research in the study area has demonstrated that wildebeest migrate into and out of the area depending on plant biomass, which is dependent on rainfall [27]. In addition, recent changes to land use in Kenya, including subdivision and fencing, have disrupted wildebeest migration patterns [28]. This could result in decreased transmission if wildebeests are prevented from returning to preferred grazing areas or increased transmission through increasing the number of non-migrating wildebeest populations close to cattle herds.

The results of this study confirm earlier reports that all age classes of cattle are susceptible to WA-MCF [5]. Steers, heifers and cows were more likely to develop WA-MCF compared to the calves in 2014, while heifers and cows were more likely to develop WA-MCF compared to the calves in 2015. This is similar to a previous study in Tanzania where 36% of respondents indicated that the disease affected mainly adults [5]. Calves were less likely to be affected than other age classes. Calves tend to be kept near the homestead and hence may not spend as much time as adults in the wildebeest grazing areas [5], and can also be suckling rather than grazing.

This study demonstrated that when laboratory confirmation of clinical diagnosis is required, nested PCR is a more effective tool for diagnosing WA-MCF than the indirect ELISA. The effectiveness of PCR has been reported previously [10]. However, the nested PCR is expensive, requiring specialized equipment and trained personnel, which means it is not easily accessible. The poor performance of the ELISA is likely due to the lack of antibody production in some cattle with WA-MCF, since susceptible animals may fail to develop measurable levels of antibody before death [12, 29]. This is reflected in the high positive predictive value of the ELISA, showing that a positive serological test is good evidence to support a diagnosis of WA-MCF whereas a negative test has no predictive value. This is also supported by the BAI which demonstrated a reasonable agreement between the indirect ELISA and the PCR in detecting positive cases and poorer agreement between the tests in detecting negative cases [30].

This study has provided a clearer understanding of the serological response to WA-MCF in a field setting. Of 123 clinically affected cattle, 77 were ELISA-positive, suggesting that almost two-thirds of cattle with PCR-confirmed WA-MCF seroconvert during the clinical course of disease. Previous research has indicated a similar serological response in cattle exposed to SA-MCF [15].

There were six ELISA-positive animals/PCR-negative animals. These results may indicate subclinical infection, recovered animals or animals infected with an antigenically cross-reactive herpesvirus. Subclinical infection of cattle with SA-MCF has been reported previously [16, 31]. However a previous serosurvey for WA-MCF in Tanzania only detected 1% seropositive cattle [32]. The role of subclinically infected animals in the epidemiology of WA-MCF in east Africa warrants further investigation.

This study highlighted the value of clinical diagnosis for confirming WA-MCF in cattle. The LCM demonstrated clinical diagnosis to have high sensitivity. Previous studies employing clinical diagnosis include a study in Northern Tanzania [33] and an outbreak of WA-MCF in Ankole cattle in a zoological park [34]. Clinical diagnosis is still the most accessible tool to the majority of cattle-keepers.

The reduced specificity of clinical diagnosis highlights the potential for non-MCF cases to be misclassified. Since some of the WA-MCF clinical signs are non-specific (fever, depression anorexia, diarrhoea and bilateral corneal opacity) there is the potential for diseases with similar clinical presentations, such as East Coast fever (ECF), to be misdiagnosed as WA-MCF [5]. These animals may be sold at reduced costs when alternative treatments might be more appropriate. The costs of WA-MCF outbreaks are direct through death of animals and loss of production. Some money can be recouped by selling acutely sick animals at 50% market value [5, 24]. The direct costs to Kapiti Plains Ranch between 2014 and 2016 were estimated to be US$97,500. The four animals classified as WA-MCF cases by clinical diagnosis but PCR and ELISA negative resulted in US$1200 loss. The sale of non-MCF cases could be avoided with improved diagnostics.

## Conclusion

The practicality of WA-MCF diagnosis in the field relies on clinical signs. This study demonstrates that clinical diagnosis is an effective method to identify affected animals. However, the possibility of incorrectly classifying animals based on clinical signs may result in unnecessary financial loss. Indirect ELISA was shown to be a poor test in comparison to nested PCR, which is a more appropriate confirmatory test for acute WA-MCF. The future of WA-MCF diagnosis should be aimed at developing penside techniques for fast detection in the field.

## Methods

### Study area

Kapiti Plains Ranch Limited is 33,800 acre ranch located in the Athi-Kaputiei plains 40 km east of Nairobi at 1.633333°S and 37.145267°E. It lies between 1646 to 1911 m above sea level. The area experiences two short rainy seasons in March–May and again in October–December. Kapiti comprises extensive open grassland and is described as a semi-arid zone. Cattle and sheep are the main livestock species farmed on the land. The livestock graze together with multiple wild herbivore species, including: wildebeest, hartebeest, Thomson’s and Grant’s gazelle, impala, zebra, ostrich, eland and giraffe. Historically, WA-MCF cases in cattle have been documented during and shortly after the wildebeest calving season from February to July.

### Study population

Kapiti Plains Ranch has between 2000 and 2500 head of cattle. These are predominantly of the Boran breed with some Boran-Friesian crosses.

### Ethical considerations

The International Livestock Research Institute (ILRI) institutional veterinarian or a technician under instruction from the veterinarian collected samples from clinically affected animals. The collection of diagnostic specimens from clinically sick animals did not require ethical review. Permission to sample animals was granted by the Kapiti Plains Ranch management and the registered resident veterinarian. Suspect cases of WA-MCF were sold for slaughter, as is the normal practice at the Kapiti Plains Ranch. Post mortem examinations were conducted on two animals that died suddenly in 2014 and one in 2016; the cause of death was confirmed to be WA-MCF. The pathological findings are not reported here. Clinically healthy animals were sampled during a concurrent vaccine study reviewed and approved by the ILRI Institutional Animal Care and Use Committee (IACUC) 2016–02; the Directorate of Veterinary Services, Republic of Kenya granted approval for the vaccine trial (VACC/I/VOL.XV/79).

### Sampling method and sample collection

Between 2014 and 2016, 325 cases of WA-MCF were reported at Kapiti Plains Ranch. Diagnosis was based on clinical signs including pyrexia, bilateral corneal opacity, serosanguinous nasal discharge and lachrymation. Additional clinical signs associated with WA-MCF were also considered but not observed in all cases. These included dyspnea/coughing, inappetence, neurological signs, salivation and weakness. Information regarding age and gender were recorded for each animal. Blood samples were opportunistically collected from 123 cases of clinically suspected WA-MCF and 51 clinically healthy animals.

Blood samples were collected by jugular venipuncture directly into 10 ml plain and 10 ml Ethylenediaminetetraacetic acid (EDTA) vacutainer tubes (Becton Dickinson). The tubes were placed in a cool box containing ice packs and transported to ILRI.

### Laboratory analysis

#### Serology

Sera were separated by centrifugation at 3000 rpm for 20 min at 4 °C (Avanti J-E Centrifuge, Beckman Coulter, USA). Sera were screened for antibodies to WA-MCF by indirect ELISA [12]. Ninety-six-well microtitre plates (Greiner Bio-One, Austria) were coated with 50 μL of 5 μg/mL of virus positive or negative ELISA antigen in 0.1 M carbonate buffer of pH 9.6. Virus-positive and negative ELISA coating antigens were produced at the Moredun Research Institute from cell-free culture fluid of bovine turbinate (BT) cells infected with attenuated AlHV-1 C500 and from uninfected BT cells respectively [12]. The plates were covered with parafilm and left overnight at 4 °C. The plates were then washed six times with phosphate buffered saline (PBS) and 0.02% Tween 20. This was followed by blocking each well with 100 μL of 4% non fat dried milk/PBS, covering with parafilm for one hour at room temperature. The plates were then washed twice with PBS/Tween 20. An aliquot of 50 μL of sample sera diluted in 2% non fat dried milk/PBS/Tween 20 (1:500) was then added in duplicate to pairs of positive and negative antigen wells. Known positive and negative serum samples were included in all plates as controls. The plates were covered with parafilm and left for 1.5 h at room temperature. The plates were then washed six times with PBS/Tween 20. This was followed with the addition of 50 μL per well of 1:1000 rabbit antibovine IgG-horseradish peroxidase (HRP) (Sigma Aldrich, USA) diluted in 2% non fat dried milk/PBS/Tween 20 and left for one hour at room temperature after covering with parafilm. The plates were washed six times with PBS/Tween 20. Tetramethylbenzidine**(**TMB) substrate (50 μL) was added to each well and colour change allowed to develop for five minutes. The reaction was stopped by adding 50 μl of 0.1 M hydrochloric acid in every well. The plates were read by at 450 nm for evaluation of the optical densities (OD) (Synergy HT, Biotek, USA). ELISA values were calculated by subtracting the mean of the negative antigen OD values from the mean of the positive antigen OD values for every sample. Inter-plate variations were adjusted by a correction factor (CF), which was applied to all of the plates that used the same positive and negative control sera over the screening period. The first plate run in a sequence with the same negative and positive controls was used in calculating the CF. The CF was calculated from the mean ELISA value of the positive and negative controls from the first plate by the equation below, which was applied to all the subsequent plates.

$$ \mathrm{Correction}\ \mathrm{factor}=\left({\mathrm{P}}_0-{\mathrm{N}}_0\right)/{\mathrm{P}}_{\mathrm{t}}-{\mathrm{N}}_{\mathrm{t}} $$

Where: P_0_ = Mean of the positive control sera from plate 1.

N_0_ = Mean of the negative control sera from plate 1.

P_t_ = Mean of the positive control sera from plate on test.

N_t_ = Mean of the negative control sera from plate on test.

The cutoff was calculated by taking the mean ELISA values of a plate of known negative samples plus three standard deviations.

#### DNA extractions

DNA was extracted from 300 μL frozen EDTA-whole blood using the Qiagen Flexigene kit (Qiagen, Venlo, Netherlands). Where frozen EDTA-whole blood samples were not available DNA was extracted from 200 μL frozen sera using the High Pure Viral Nucleic Acid kit (Roche Life Sciences, Basel, Switzerland) DNA was extracted according to the manufacturer’s instructions and eluted in 100 μL elution buffer and stored at minus 20 °C.

#### Nested PCR

Amplification reactions were performed using previously described primers [21]. The PCR reaction mix was 25 μL and contained 12.5 μL of oneTaq Universal Master Mix (New England Biolabs; containing OneTaq® DNA polymerase, dNTPs, buffer components), 5 pmol forward primer, 5 pmol reverse primer and a minimum of 50 ng of sample DNA. The initial round of nested PCR was performed using outer forward and outer reverse primers, C500–1: TACGGGTGCCCTGACATTTCATCTCTTTTG; and C500–2: ATAACTGGTTGATGTGGCAGATGCATCTAT respectively.

The second round amplification (274 basepair (bp) DNA fragments) used the first round product (2 μL), with inner forward and inner reverse primers, C500–3: TCTGGCCCGTGCTGCAGCAAGACTCTCAG; and C500–4: TATAGTAGAATCCCGTCTGAGTGGTAGCTG.

Thermocycling conditions for both rounds of amplification were: 95 °C for 5 min; followed by 34 cycles of 94 °C for 1 min; 55 °C for 1 min; 72 °C for 2 min; and 72 °C for 7 min. PCR products (6 μL) were electrophoresed at 80 V through 2.0% (*w*/*v*) agarose containing 4 μL GelRed (Biotium, 10,000x) per 100 ml gel. Gels were photographed by ultraviolet light for PCR product analysis. The positive control was DNA extracted from the blood of cattle that were confirmed to have died of WA-MCF. Sterile water served as negative controls.

### Statistical analysis

Data was managed in Microsoft (MS) Excel (Microsoft Corp., 2010). Descriptive statistics and graphs were produced in MS Excel. Prevalence estimates were calculated using the *trueprev* function in the *prevalence* package [35] in R environment for statistical computing, version 3.4.0 (http://cran.r-project.org/). The logistic regression analysis was conducted using the *glm* function in the base package of R. Odds ratios (OR) were calculated and *P* values ≤0.05 were considered significant. Estimate of financial losses were calculated as 50% of the value of a mature Boran animal (US$600) multiplied by the number of animals disposed/sold due to WA-MCF in each year.

### Diagnostic test performance

Comparison between the diagnostic tests was conducted using four methods.

1. The Cohen kappa statistic was used to assess the agreement between PCR and indirect ELISA with scores divided into < 0.2: slight agreement, 0.2–0.4: fair agreement, 0.4–0.6: moderate agreement, 0.6–0.8: substantial agreement and > 0.8: almost perfect agreement [36].
2. The relative sensitivity (Se) and specificity (Sp) of indirect ELISA was calculated using the nested PCR as a gold standard [36]**.**
3. The Bayesian Agreement Index (BAI) framework was also performed in the R software environment to determine the level of agreement between ELISA and PCR in the absence of a gold standard [37, 38]
4. A Hui-Walter latent class model was used to estimate the sensitivity and specificity of clinical diagnoses, nested PCR and indirect ELISA in the absence of a gold-standard [39]. The model was adapted for three tests in 1 population [40]. The latent class approach assumes that none of the tests is a “gold standard” [41] and the tests are conditionally independent, such that if the true disease status of an animal is known, then the outcome of one test does not change our confidence in the outcome of the other test [40].

- As previously described by Bronsvoort et al. (2006) the Bayesian approach to the Hui-Walter model assumes that for the *i*th subpopulation the tally (O*i*) of the different amalgam of test results for three tests +/+/+, +/+/−, +/−/+, etc., follow a multinomial distribution, where Pr_*i*_ is a vector of probabilities of observing the individual combinations of test results, S is number of subpopulations, and T is the number of tests and:

$$ {O}_i\mid {Se}_j{Sp}_j{p}_i\sim Multinomial\left({\mathit{\Pr}}_i,{n}_i\right) for\ i=1,2,\dots ..,S\  and\ j=1,2,\dots .,T $$

- Probabilities for each test scenario can be derived using the Se/Sp of the tests and prevalence (p) of the population. The equation below is for three positive tests:

$$ \Pr \left({T}_1+,{T}_2+,{T}_3+\right)={Se}_1{Se}_2{Se}_3{p}_i+\left(1-{Sp}_1\right)\left(1-{Sp}_2\right)\left(1-{Sp}_3\right)\left(1-{p}_i\right) $$

- Estimates of test sensitivity and specificity were incorporated into the Bayesian framework as priors to inform posterior estimates. Uninformed, beta(1,1) priors were given to the estimates for sensitivity and specificity of clinical diagnosis and ELISA and beta(5,1) priors for PCR. The sensitivity and specificity of PCR for detecting WA-MCF has been reported to be 95–97% and 94–100% respectively [42].
- The model was built using the *runjags* package [43] to call Just another Gibbs sampler (JAGS) software [44] in R. A burn in period of 50,000 iterations was discarded and every tenth iteration of the following 100,000 kept for posterior inference [40]. Convergence was assessed by visual inspection of the time-series plots and Gelman-Rubin diagnostic plots using three chains with different starting points [40]. Posterior means and 95% credibility intervals were calculated for the Se and Sp of the three tests.

## Additional files

Additional file 1: Descriptive results of WA-MCF outbreaks at Kapiti Plains Ranch from 2014 to 2016. This table shows the number of WA-MCF cases between 2014 and 2016 classified by age, sex and breed. (DOCX 15 kb)

Additional file 2: Results of the logistic regression analysis demonstrating the relationship between WA-MCF infection, gender and age group during outbreaks at Kapiti Plains Ranch 2014–2016. This table shows the results of the logistic regression analysis to measure associations between age, sex, breed and WA-MCF seropositivity (DOCX 17 kb)

Additional file 3: Details for each animal sampled at Kapiti Plains Ranch 2014–2016 - identification, date of birth, sex, sampling date, breed, samples available, clinical status and test results. Column headings: Brand, animal identification; DOB, date of birth; Sex, M = male, F = female; Sample date, date sample collected; Breed, Boran = Boran breed, Dairy = Boran cross Friesian or Ayrshire; Blood, Yes = blood sample available for testing, No = sample not available; Serum, Yes = serum sample available for testing, No = sample not available; Clinical, Yes = animal presented with clinical WA-MCF, No = animal did not have clinical signs; PCR, Positive = positive test result, Negative = negative in all tests; ELISA, Positive = positive ELISA value, Negative = negative in all tests. (DOCX 43 kb)

## Abbreviations

AlHV-1: Alcelaphine herpesvirus 1
BAI: Bayesian Agreement Index
BCI: Bayesian Credibility Interval
bp: Base pair
BT: Bovine turbinate
BVD: Bovine viral diarrhoea
CF: Correction factor
CI-: Competitive inhibition
CI: Confidence Interval
DNA: Deoxyribonucleic acid
dNTPs: deoxyribonucleotide triphosphates
ECF: East coast fever
EDTA: Ethylenediaminetetraacetic acid
ELISA: Enzyme linked immunosorbent assay
FMD: Foot and mouth disease
HRP: Horseradish peroxidase
IACUC: Institutional Animal Care and Use Committee
IgG: Immunoglobulin gamma
ILRI: International Livestock Research Institute
JAGS: Just another Gibbs sampler
LCM: Latent class model
MCF: Malignant catarrhal fever
MS: Microsoft
OD: Optical densities
OIE: World Organisation for Animal Health
OR: Odds ratios
OvHV-2: Ovine herpesvirus 2
PBS: Phosphate buffered saline
PCI: Posterior Credibility Intervals
PCR: Polymerase chain reaction
SA-MCF: Sheep associated malignant catarrhal fever
Se: Sensitivity
Sp: Specificity
TMB: Tetramethylbenzidine
US$: United States dollar
VNT: Virus neutralization test
WA-MCF: Wildebeest associated malignant catarrhal fever
μL: microlitre
