# Supplementary material for: Field validation of clinical and laboratory diagnosis of wildebeest associated malignant catarrhal fever in cattle
Source: BMC Vet Res. 2019 Feb 28;15:69. doi: 10.1186/s12917-019-1818-8 (PMC6396541; doi:10.1186/s12917-019-1818-8)
Supplement: Supplementary file 1 — Descriptive results of WA-MCF outbreaks at Kapiti Plains Ranch from 2014 to 2016. This table shows the number of WA-MCF cases between 2014 and 2016 classified by age, sex and breed. (DOCX 15 kb) [file 12917_2019_1818_MOESM1_ESM.docx]

| **Additional File 1 Descriptive results of WA-MCF outbreaks at Kapiti Plains Ranch from 2014 to 2016** | | | | | | | | | | |
| --- | --- | --- | --- | --- | --- | --- | --- | --- | --- | --- |
| **Year** | **Cases/**  **Susceptible**  **%**  **(95% C.I.)** | **Gender (excluding calves)** | | **Breed (excluding steers)** | | **Age structure** | | | | |
|  |  | Male | Female | Boran | Other breeds | Calves  <10months | Steers  10-48 months | Heifers  10-36 months | Bulls | Cows  >36 months |
| 2014 | 215/1975  8.7%  (7.6-9.9) | 54/459  11.8%  (9.1-15.0) | 152/1228  11.8%  (10.1-13.7) | 160/1462  10.9%  (9.4-12.7) | 3/182  1.6%  (0.6-4.6) | 9/228  3.9%  (2.1-7.4) | 52/331  15.7%  (12.2-19.9) | 75/578  13.0%  (10.5-15.9) | 2/128  1.6%  (0.5-5.5) | 73/710  10.3%  (8.3-12.7) |
| 2015 | 78/2106  3.7%  (3.0-4.6) | 7/398  1.8%  (0.9-3.6) | 60/1328  4.5%  (3.5-5.7) | 71/1618  4.4%  (3.5-5.5) | 1/234  0.4%  (0.1-2.5) | 11/380  2.9%  (1.7-5.1) | 6/254  2.4%  (1.1-5.1) | 28/574  4.9%  (3.4-6.9) | 1/144  0.7%  (0.2-3.8) | 32/754  4.2%  (3.1-5.9) |
| 2016 | 32/2069  1.5%  (1.1-2.2) | 4/481  0.8%  (0.3-2.1) | 26/1406  1.8%  (1.3-2.7) | 31/1482  0.2%  (0.1-0.3) | 0/227  0%  (0.0-1.6) | 2/182  1.1%  (0.3-3.9) | 1/360  0.5%  (0.1-3.0) | 1/625  0.3%  (0.1-1.6) | 3/121  0.5%  (0.9-6.8) | 25/781  3.2%  (2.2-4.7) |
