# Supplementary material for: Field validation of clinical and laboratory diagnosis of wildebeest associated malignant catarrhal fever in cattle
Source: BMC Vet Res. 2019 Feb 28;15:69. doi: 10.1186/s12917-019-1818-8 (PMC6396541; doi:10.1186/s12917-019-1818-8)
Supplement: Supplementary file 2 — Results of the logistic regression analysis demonstrating the relationship between WA-MCF infection, gender and age group during outbreaks at Kapiti Plains Ranch 2014–2016. This table shows the results of the logistic regression analysis to measure associations between age, sex, breed and WA-MCF seropositivity (DOCX 17 kb) [file 12917_2019_1818_MOESM2_ESM.docx]

| **Year** | **Variable** | | **Totals** | **Positives** | **Odds ratio** | **95% C.I** | | **P value** |
| --- | --- | --- | --- | --- | --- | --- | --- | --- |
| **2014** | Sex | Male | 459 | 54 | 1 | (0.71-1.37) | | 0.98 |
|  |  | Female | 1288 | 152 | 1 |  | | reference |
|  | Age group | Calves | 228 | 9 | 1 |  | | reference |
|  |  | Cows | 710 | 73 | 2.79 | (1.44-6.07) | | 0.005 |
|  |  | Heifers | 578 | 79 | 3.85 | (2.00-8.37) | | 0 |
|  |  | Steers | 331 | 52 | 4.53 | (2.29-10.03) | | 0.00005 |
|  |  | Bulls | 128 | 2 | 0.39 | (0.05-1.53) | | 0.228 |
| **2015** | Sex | Male | 398 | 7 | 0.38 | (0.15-0.77) | 0.016 | |
|  |  | Female | 1328 | 60 | 1 |  | reference | |
|  | Age group | Calves | 380 | 11 | 1 |  | reference | |
|  |  | Cows | 754 | 32 | 1.49 | (0.76-3.11) | 0.264 | |
|  |  | Heifers | 574 | 28 | 1.72 | (0.86-3.64) | 0.134 | |
|  |  | Steers | 254 | 6 | 0.81 | (0.27-2.16) | 0.685 | |
|  |  | Bulls | 144 | 1 | 0.23 | (0.01-1.22) | 0.167 | |
| **2016** | Sex | Male | 481 | 4 | 0.45 | (0.13-1.15) | 0.134 | |
|  |  | Female | 1406 | 26 | 1 | - | reference | |
|  | Age group | Calves | 182 | 2 | 1 | - | reference | |
|  |  | Cows | 781 | 25 | 2.98 | 0.88-8.60) | 0.14 | |
|  |  | Heifers | 625 | 1 | 0.14 | 0.01-1.51 | 0.115 | |
|  |  | Steers | 360 | 1 | 0.25 | (0.01-2.63) | 0.26 | |
|  |  | Bulls | 121 | 3 | 2.29 | (0.37-7.57) | 0.369 | |

**Additional File 2**

**Results of the logistic regression analysis demonstrating the relationship between WA-MCF infection, gender and age group during outbreaks at Kapiti Plains Ranch 2014-2016**
