# Supplementary material for: Field validation of clinical and laboratory diagnosis of wildebeest associated malignant catarrhal fever in cattle
Source: BMC Vet Res. 2019 Feb 28;15:69. doi: 10.1186/s12917-019-1818-8 (PMC6396541; doi:10.1186/s12917-019-1818-8)
Supplement: Supplementary file 3 — Details for each animal sampled at Kapiti Plains Ranch 2014–2016 - identification, date of birth, sex, sampling date, breed, samples available, clinical status and test results. Column headings: Brand, animal identification; DOB, date of birth; Sex, M = male, F = female; Sample date, date sample collected; Breed, Boran = Boran breed, Dairy = Boran cross Friesian or Ayrshire; Blood, Yes = blood sample available for testing, No = sample not available; Serum, Yes = serum sample available for testing, No = sample not available; Clinical, Yes = animal presented with clinical WA-MCF, No = animal did not have clinical signs; PCR, Positive = positive test result, Negative = negative in all tests; ELISA, Positive = positive ELISA value, Negative = negative in all tests. (DOCX 43 kb) [file 12917_2019_1818_MOESM3_ESM.docx]

Additional File 3 Details for each animal sampled at Kapiti Plains Ranch 2014-2016 - identification, date of birth, sex, sampling date, breed, samples available, clinical status and test results

Column headings: Brand, animal identification; DOB, date of birth; Sex, M= male, F= female; Sample date, date sample collected; Breed, Boran=Boran breed, Dairy = Boran cross Friesian or Ayrshire; Blood, Yes= blood sample available for testing, No = sample not available; Serum, Yes= serum sample available for testing, No= sample not available; Clinical, Yes = animal presented with clinical WA-MCF, No = animal did not have clinical signs; PCR, Positive=positive test result, Negative=negative in all tests; ELISA, Positive=positive ELISA value, Negative=negative in all tests.

| **BRAND** | **DOB** | **SEX** | **Sample date** | **Breed** | **Blood** | **Serum** | **Clinical** | **PCR** | **ELISA** |
| --- | --- | --- | --- | --- | --- | --- | --- | --- | --- |
| 2132 | 11/05/12 | M | 15/05/14 | Boran | No | Yes | Yes | Positive | Negative |
| 2203 | 09/06/12 | M | 15/05/14 | Dairy | No | Yes | Yes | Positive | Negative |
| 1597 | 13/07/11 | M | 15/05/14 | Dairy | No | Yes | Yes | Positive | Negative |
| 2607 | 13/04/13 | M | 15/05/14 | Boran | No | Yes | Yes | Positive | Negative |
| 2648 | 04/05/13 | M | 15/05/14 | Boran | No | Yes | Yes | Positive | Positive |
| 2564 | 17/03/13 | M | 15/05/14 | Boran | No | Yes | Yes | Negative | Negative |
| 2396 | 02/11/12 | M | 15/05/14 | Boran | No | Yes | Yes | Positive | Positive |
| 1590 | 03/07/11 | M | 15/05/14 | Boran | No | Yes | Yes | Positive | Negative |
| D780 | 01/01/02 | F | 15/05/14 | Dairy | No | Yes | Yes | Positive | Negative |
| 220 | 29/04/08 | F | 15/05/14 | Boran | No | Yes | Yes | Positive | Positive |
| 40206F | 10/06/06 | F | 15/05/14 | Boran | No | Yes | Yes | Positive | Positive |
| 08104B | 06/02/04 | F | 15/05/14 | Boran | No | Yes | Yes | Positive | Positive |
| 706 | 28/02/03 | F | 15/05/14 | Boran | No | Yes | Yes | Positive | Positive |
| 2259 | 13/07/12 | F | 15/05/14 | Boran | No | Yes | Yes | Positive | Positive |
| 2226 | 17/06/12 | F | 15/05/14 | Boran | No | Yes | Yes | Positive | Negative |
| 2168 | 27/05/12 | F | 15/05/14 | Boran | No | Yes | Yes | Positive | Negative |
| 2045 | 04/04/12 | F | 15/05/14 | Boran | No | Yes | Yes | Positive | Positive |
| 2481 | 10/01/13 | F | 15/05/14 | Boran | No | Yes | Yes | Positive | Positive |
| 2395 | 02/11/12 | F | 15/05/14 | Boran | No | Yes | Yes | Positive | Positive |
| 2239 | 23/06/12 | F | 15/05/14 | Boran | No | Yes | Yes | Negative | Positive |
| 2152 | 20/05/12 | F | 15/05/14 | Boran | No | Yes | Yes | Positive | Negative |
| 2022 | 25/03/12 | F | 15/05/14 | Boran | No | Yes | Yes | Negative | Positive |
| 2470 | 01/01/13 | F | 15/05/14 | Boran | No | Yes | Yes | Positive | Positive |
| 2146 | 17/05/12 | F | 15/05/14 | Boran | No | Yes | Yes | Positive | Positive |
| 2398 | 03/11/12 | F | 18/05/14 | Boran | No | Yes | Yes | Positive | Positive |
| 1618 | 09/08/11 | M | 21/05/14 | Boran | Yes | Yes | Yes | Positive | Negative |
| 2475 | 05/01/13 | M | 21/05/14 | Boran | Yes | Yes | Yes | Positive | Negative |
| 410 | 08/07/08 | F | 25/05/14 | Boran | Yes | Yes | Yes | Positive | Negative |
| 408 | 22/06/07 | F | 25/05/14 | Boran | Yes | Yes | Yes | Positive | Negative |
| 650 | 22/01/09 | F | 25/05/14 | Boran | Yes | Yes | Yes | Positive | Positive |
| 296 | 28/05/08 | F | 25/05/14 | Boran | Yes | Yes | Yes | Positive | Positive |
| 779 | 28/04/09 | M | 25/05/14 | Boran | Yes | Yes | Yes | Positive | Positive |
| 3039 | 12/01/14 | F | 25/05/14 | Boran | Yes | Yes | Yes | Positive | Positive |
| 2350 | 30/09/12 | F | 25/05/14 | Boran | Yes | Yes | Yes | Positive | Positive |
| 262 | 21/05/08 | F | 25/05/14 | Boran | Yes | Yes | Yes | Positive | Negative |
| 1971 | 02/03/12 | F | 25/05/14 | Boran | Yes | Yes | Yes | Positive | Positive |
| 2006 | 16/03/12 | F | 25/05/14 | Boran | Yes | Yes | Yes | Positive | Positive |
| 2210 | 11/06/12 | M | 25/05/14 | Boran | Yes | Yes | Yes | Positive | Positive |
| 656 | 24/06/05 | F | 28/05/14 | Boran | No | Yes | Yes | Positive | Positive |
| 476 | 09/08/07 | F | 28/05/14 | Boran | No | Yes | Yes | Positive | Positive |
| 29406E | 05/05/06 | F | 28/05/14 | Boran | No | Yes | Yes | Positive | Positive |
| 1666 | 25/09/11 | M | 28/05/14 | Boran | No | Yes | Yes | Positive | Positive |
| 2472 | 02/01/13 | M | 28/05/14 | Boran | No | Yes | Yes | Positive | Negative |
| 2171 | 30/05/12 | F | 28/05/14 | Boran | No | Yes | Yes | Positive | Negative |
| 2449 | 15/12/12 | F | 28/05/14 | Boran | No | Yes | Yes | Positive | Positive |
| 2301 | 26/08/12 | F | 28/05/14 | Boran | Yes | Yes | Yes | Positive | Negative |
| 2030 | 26/03/12 | F | 28/05/14 | Boran | Yes | Yes | Yes | Positive | Negative |
| 2025 | 25/03/12 | F | 28/05/14 | Boran | No | Yes | Yes | Positive | Negative |
| 2505 | 01/02/13 | M | 28/05/14 | Boran | No | Yes | Yes | Positive | Negative |
| 17904C | 17/04/04 | F | 29/05/14 | Boran | No | Yes | Yes | Positive | Positive |
| 1661 | 21/09/11 | M | 29/05/14 | Boran | No | Yes | Yes | Positive | Negative |
| 2040 | 01/04/12 | F | 29/05/14 | Boran | No | Yes | Yes | Positive | Negative |
| 2362 | 08/10/12 | F | 29/05/14 | Boran | No | Yes | Yes | Positive | Positive |
| 2357 | 07/10/12 | F | 29/05/14 | Boran | No | Yes | Yes | Positive | Positive |
| 2258 | 13/07/12 | F | 29/05/14 | Boran | No | Yes | Yes | Positive | Negative |
| 1904 | 13/02/12 | F | 31/05/14 | Boran | No | Yes | Yes | Positive | Positive |
| 1992 | 10/03/12 | F | 31/05/14 | Boran | No | Yes | Yes | Positive | Negative |
| 2438 | 29/11/12 | F | 31/05/14 | Boran | Yes | Yes | Yes | Positive | Negative |
| 2299 | 25/08/12 | F | 31/05/14 | Boran | Yes | Yes | Yes | Positive | Positive |
| 1566 | 07/06/11 | M | 31/05/14 | Boran | Yes | Yes | Yes | Positive | Negative |
| 2286 | 11/08/12 | M | 31/05/14 | Boran | Yes | Yes | Yes | Positive | Negative |
| 1757 | 15/12/11 | M | 31/05/14 | Boran | Yes | Yes | Yes | Positive | Negative |
| 2304 | 31/08/12 | F | 02/06/14 | Boran | No | Yes | Yes | Positive | Negative |
| 2443 | 06/12/12 | F | 02/06/14 | Boran | No | Yes | Yes | Positive | Positive |
| 2451 | 15/12/12 | M | 02/06/14 | Boran | No | Yes | Yes | Positive | Positive |
| 965 | 01/12/09 | F | 03/06/14 | Boran | No | Yes | Yes | Positive | Negative |
| 670 | 21/11/07 | F | 03/06/14 | Boran | No | Yes | Yes | Positive | Positive |
| 2024 | 25/03/12 | F | 03/06/14 | Boran | No | Yes | Yes | Negative | Negative |
| 2122 | 08/05/12 | M | 03/06/14 | Dairy | No | Yes | Yes | Negative | Negative |
| 2329 | 18/09/12 | M | 05/06/14 | Boran | No | Yes | Yes | Positive | Negative |
| 2354 | 05/10/12 | M | 05/06/14 | Boran | Yes | Yes | Yes | Positive | Negative |
| 2065 | 15/04/12 | F | 06/06/14 | Boran | Yes | Yes | Yes | Positive | Negative |
| 2322 | 14/09/12 | F | 06/06/14 | Boran | Yes | Yes | Yes | Positive | Positive |
| 579 | 01/10/07 | F | 07/06/14 | Boran | Yes | Yes | Yes | Positive | Positive |
| 2313 | 07/09/12 | F | 07/06/14 | Boran | Yes | Yes | Yes | Positive | Negative |
| 2931 | 25/09/13 | M | 07/06/14 | Dairy | Yes | Yes | Yes | Positive | Positive |
| 58805J | 28/10/05 | F | 08/06/14 | Boran | Yes | Yes | Yes | Positive | Positive |
| 474 | 09/08/07 | F | 08/06/14 | Boran | Yes | Yes | Yes | Positive | Positive |
| 3063 | 02/02/14 | F | 08/06/14 | Dairy | Yes | Yes | Yes | Positive | Positive |
| 3075 | 13/02/14 | F | 26/05/14 | Boran | No | Yes | Yes | Negative | Negative |
| 348-07 | 01/05/07 | F | 18/04/15 | Boran | Yes | Yes | Yes | Positive | Negative |
| 1276 | 14/01/11 | F | 18/04/15 | Boran | Yes | Yes | Yes | Positive | Negative |
| 370 | 19/06/08 | F | 20/04/15 | Boran | Yes | Yes | Yes | Positive | Positive |
| 2879 | 19/08/13 | F | 20/04/15 | Boran | Yes | Yes | Yes | Positive | Positive |
| 2864 | 10/08/13 | F | 20/04/15 | Boran | Yes | Yes | Yes | Positive | Negative |
| 2889 | 26/08/13 | F | 20/04/15 | Boran | Yes | Yes | Yes | Positive | Positive |
| 2592 | 06/04/13 | F | 20/04/15 | Boran | Yes | Yes | Yes | Positive | Positive |
| 3511 | 29/11/14 | M | 21/04/15 | Boran | Yes | Yes | Yes | Positive | Positive |
| 1288 | 18/01/11 | F | 23/04/15 | Boran | Yes | Yes | Yes | Positive | Positive |
| 2862 | 09/08/13 | F | 24/04/15 | Boran | Yes | Yes | Yes | Positive | Positive |
| 3574 | 29/12/14 | M | 24/04/15 | Boran | Yes | Yes | Yes | Positive | Positive |
| 3562 | 18/12/14 | M | 29/03/16 | Boran | Yes | Yes | Yes | Positive | Negative |
| 513 | 01/09/07 | F | 06/05/16 | Boran | Yes | Yes | Yes | Negative | Positive |
| 683 | 27/02/09 | F | 07/05/16 | Boran | Yes | Yes | Yes | Positive | Positive |
| 4074 | 28/12/15 | M | 09/05/16 | Boran | Yes | Yes | Yes | Positive | Positive |
| 218 | 28/04/08 | F | 18/05/16 | Boran | Yes | Yes | Yes | Positive | Positive |
| 666 | 07/02/09 | F | 25/05/16 | Boran | Yes | Yes | Yes | Positive | Positive |
| 299 | 01/05/07 | F | 27/05/16 | Boran | Yes | Yes | Yes | Positive | Positive |
| 07105B | 01/02/05 | F | 30/05/16 | Boran | Yes | Yes | Yes | Positive | Positive |
| 457 | 06/09/08 | F | 06/06/16 | Boran | Yes | Yes | Yes | Positive | Positive |
| 4001 | 03/10/05 | M | 07/06/16 | Boran | Yes | Yes | Yes | Positive | Positive |
| 1458 | 28/03/11 | F | 11/06/16 | Boran | Yes | Yes | Yes | Positive | Positive |
| 1309 | 26/01/11 | F | 18/06/16 | Boran | Yes | Yes | Yes | Positive | Positive |
| 1474 | 06/04/11 | M | 21/06/16 | Boran | Yes | Yes | Yes | Positive | Positive |
| 2742 | 01/05/13 | F | 23/06/16 | Boran | Yes | Yes | Yes | Positive | Positive |
| 153 | 26/03/08 | F | 28/06/16 | Boran | Yes | Yes | Yes | Positive | Positive |
| 1058 | 20/05/10 | F | 28/06/16 | Boran | Yes | Yes | Yes | Positive | Positive |
| 688 | 03/03/09 | F | 02/07/16 | Boran | Yes | Yes | Yes | Positive | Positive |
| 673 | 01/12/05 | F | 02/07/16 | Boran | Yes | Yes | Yes | Positive | Positive |
| 335 | 08/06/08 | F | 02/07/16 | Boran | Yes | Yes | Yes | Positive | Negative |
| 358 | 01/05/07 | F | 02/07/16 | Boran | Yes | Yes | Yes | Positive | Positive |
| 1822 | 12/01/12 | F | 02/07/16 | Boran | Yes | Yes | Yes | Positive | Positive |
| 1149 | 12/11/10 | M | 02/07/16 | Boran | Yes | Yes | Yes | Positive | Negative |
| 267 | 23/05/08 | F | 03/07/16 | Boran | Yes | Yes | Yes | Positive | Positive |
| 15407 | 01/03/07 | F | 04/07/16 | Boran | Yes | Yes | Yes | Positive | Positive |
| 1665 | 25/09/11 | F | 05/07/16 | Boran | Yes | Yes | Yes | Positive | Positive |
| 508 | 01/09/07 | F | 09/07/16 | Boran | Yes | Yes | Yes | Positive | Positive |
| 309 | 01/06/08 | F | 10/07/16 | Boran | Yes | Yes | Yes | Positive | Positive |
| 725 | 24/03/09 | F | 18/07/16 | Boran | Yes | Yes | Yes | Positive | Positive |
| 1126 | 26/10/10 | F | 23/07/16 | Boran | Yes | Yes | Yes | Positive | Positive |
| 3745 | 13/03/15 | F | 29/07/16 | Boran | Yes | Yes | Yes | Positive | Negative |
| 4302 | 17/06/16 | F | 20/08/16 | Boran | Yes | Yes | Yes | Positive | Negative |
| 748 | 07/04/09 | F | 18/09/16 | Boran | Yes | Yes | Yes | Positive | Positive |
| 3344 | 10/08/14 | M | 23/05/16 | Dairy | Yes | Yes | No | Negative | Negative |
| 3371 | 16/09/14 | M | 23/05/16 | Boran | Yes | Yes | No | Negative | Negative |
| 3375 | 17/09/14 | M | 23/05/16 | Boran | Yes | Yes | No | Negative | Negative |
| 3379 | 19/09/14 | M | 23/05/16 | Boran | Yes | Yes | No | Negative | Negative |
| 3402 | 01/10/14 | M | 23/05/16 | Dairy | Yes | Yes | No | Negative | Positive |
| 3415 | 12/10/14 | M | 23/05/16 | Boran | Yes | Yes | No | Negative | Negative |
| 3436 | 20/10/14 | M | 23/05/16 | Boran | Yes | Yes | No | Negative | Negative |
| 3465 | 04/11/14 | M | 23/05/16 | Boran | Yes | Yes | No | Negative | Negative |
| 3475 | 11/11/14 | M | 23/05/16 | Boran | Yes | Yes | No | Negative | Negative |
| 3498 | 25/11/14 | M | 23/05/16 | Dairy | Yes | Yes | No | Negative | Negative |
| 3542 | 12/12/14 | M | 23/05/16 | Boran | Yes | Yes | No | Negative | Positive |
| 3571 | 25/12/14 | M | 23/05/16 | Boran | Yes | Yes | No | Negative | Negative |
| 3602 | 10/01/15 | M | 23/05/16 | Boran | Yes | Yes | No | Negative | Negative |
| 3612 | 17/01/15 | M | 23/05/16 | Dairy | Yes | Yes | No | Negative | Negative |
| 3624 | 22/01/15 | M | 23/05/16 | Boran | Yes | Yes | No | Negative | Negative |
| 3631 | 25/01/15 | M | 23/05/16 | Boran | Yes | Yes | No | Negative | Negative |
| 3635 | 25/01/15 | M | 23/05/16 | Boran | Yes | Yes | No | Negative | Negative |
| 3656 | 31/01/15 | M | 23/05/16 | Dairy | Yes | Yes | No | Negative | Negative |
| 3664 | 03/02/15 | M | 23/05/16 | Boran | Yes | Yes | No | Negative | Negative |
| 3719 | 27/02/15 | M | 23/05/16 | Boran | Yes | Yes | No | Negative | Negative |
| 3774 | 29/03/15 | M | 23/05/16 | Dairy | Yes | Yes | No | Negative | Negative |
| 3807 | 30/04/15 | M | 23/05/16 | Boran | Yes | Yes | No | Negative | Negative |
| 3814 | 07/05/15 | M | 23/05/16 | Boran | Yes | Yes | No | Negative | Negative |
| 3816 | 07/05/15 | M | 23/05/16 | Boran | Yes | Yes | No | Negative | Negative |
| 3823 | 11/05/15 | M | 23/05/16 | Boran | Yes | Yes | No | Negative | Negative |
| 3831 | 16/05/15 | M | 23/05/16 | Boran | Yes | Yes | No | Negative | Negative |
| 3310 | 21/06/14 | M | 23/05/16 | Boran | Yes | Yes | No | Negative | Negative |
| 3365 | 06/09/14 | M | 23/05/16 | Boran | Yes | Yes | No | Negative | Negative |
| 3381 | 20/09/14 | M | 23/05/16 | Boran | Yes | Yes | No | Negative | Negative |
| 3425 | 16/10/14 | M | 23/05/16 | Boran | Yes | Yes | No | Negative | Negative |
| 3426 | 16/10/14 | M | 23/05/16 | Boran | Yes | Yes | No | Negative | Negative |
| 3463 | 02/11/14 | M | 23/05/16 | Boran | Yes | Yes | No | Negative | Negative |
| 3472 | 09/11/14 | M | 23/05/16 | Dairy | Yes | Yes | No | Negative | Negative |
| 3496 | 23/11/14 | M | 23/05/16 | Boran | Yes | Yes | No | Negative | Negative |
| 3529 | 06/12/14 | M | 23/05/16 | Boran | Yes | Yes | No | Negative | Negative |
| 3554 | 15/12/14 | M | 23/05/16 | Dairy | Yes | Yes | No | Negative | Negative |
| 3555 | 16/12/14 | M | 23/05/16 | Boran | Yes | Yes | No | Negative | Negative |
| 3567 | 22/12/14 | M | 23/05/16 | Dairy | Yes | Yes | No | Negative | Positive |
| 3604 | 12/01/15 | M | 23/05/16 | Dairy | Yes | Yes | No | Negative | Negative |
| 3609 | 16/01/15 | M | 23/05/16 | Boran | Yes | Yes | No | Negative | Negative |
| 3617 | 18/01/15 | M | 23/05/16 | Boran | Yes | Yes | No | Negative | Negative |
| 3626 | 22/01/15 | M | 23/05/16 | Dairy | Yes | Yes | No | Negative | Negative |
| 3693 | 14/02/15 | M | 23/05/16 | Boran | Yes | Yes | No | Negative | Negative |
| 3699 | 16/02/15 | M | 23/05/16 | Boran | Yes | Yes | No | Negative | Negative |
| 3737 | 11/03/15 | M | 23/05/16 | Dairy | Yes | Yes | No | Negative | Negative |
| 3790 | 13/04/15 | M | 23/05/16 | Dairy | Yes | Yes | No | Negative | Negative |
| 3833 | 20/05/15 | M | 23/05/16 | Boran | Yes | Yes | No | Negative | Negative |
| 3835 | 20/05/15 | M | 23/05/16 | Dairy | Yes | Yes | No | Negative | Negative |
| 3841 | 23/05/15 | M | 23/05/16 | Boran | Yes | Yes | No | Negative | Negative |
| 3844 | 27/05/15 | M | 23/05/16 | Boran | Yes | Yes | No | Negative | Negative |
| 3787 | 08/04/15 | M | 23/05/16 | Boran | Yes | Yes | No | Negative | Negative |
